# *Microsporum canis* complex infections in the United Arab Emirates: Clinical, molecular, and antifungal susceptibility profiles

**DOI:** 10.1093/mmy/myag091

**Published:** 2026-08-29

**Authors:** Febin Anes, Akela Ghazawi, Hari Pankaj Vanam, Dana Aljneibi, Mushtaq Khan, Fouzia Jabeen, Ahmed R Alsuwaidi, Jens Thomsen, Mohammad AlBataineh, Stefan Weber, Connie Gibas, Nathan P Wiederhold, Fatima Al Dhaheri

**Affiliations:** Department of Pediatrics and Department of Medical Microbiology and Immunology, College of Medicine and Health Sciences, Al Ain, Abu Dhabi 15551, United Arab Emirates; Zayed Bin Sultan Centre for Health Sciences, United Arab Emirates University, Al Ain, Abu Dhabi 15551, United Arab Emirates; Department of Pediatrics and Department of Medical Microbiology and Immunology, College of Medicine and Health Sciences, Al Ain, Abu Dhabi 15551, United Arab Emirates; Zayed Bin Sultan Centre for Health Sciences, United Arab Emirates University, Al Ain, Abu Dhabi 15551, United Arab Emirates; Department of Pediatrics and Department of Medical Microbiology and Immunology, College of Medicine and Health Sciences, Al Ain, Abu Dhabi 15551, United Arab Emirates; Zayed Bin Sultan Centre for Health Sciences, United Arab Emirates University, Al Ain, Abu Dhabi 15551, United Arab Emirates; Department of Pediatrics and Department of Medical Microbiology and Immunology, College of Medicine and Health Sciences, Al Ain, Abu Dhabi 15551, United Arab Emirates; Zayed Bin Sultan Centre for Health Sciences, United Arab Emirates University, Al Ain, Abu Dhabi 15551, United Arab Emirates; Department of Pediatrics and Department of Medical Microbiology and Immunology, College of Medicine and Health Sciences, Al Ain, Abu Dhabi 15551, United Arab Emirates; Zayed Bin Sultan Centre for Health Sciences, United Arab Emirates University, Al Ain, Abu Dhabi 15551, United Arab Emirates; Sheikh Khalifa Medical City, PureLab, Abu Dhabi 51900, United Arab Emirates; Department of Pediatrics and Department of Medical Microbiology and Immunology, College of Medicine and Health Sciences, Al Ain, Abu Dhabi 15551, United Arab Emirates; Zayed Bin Sultan Centre for Health Sciences, United Arab Emirates University, Al Ain, Abu Dhabi 15551, United Arab Emirates; Medics Labor AG, Bern 3001, Switzerland; Institute of Medical Microbiology, University of Zürich 8028, Switzerland; Khalifa University, Abu Dhabi 127788, United Arab Emirates; Yarmouk University, Irbid 21163, Jordan; Sheikh Khalifa Medical City, PureLab, Abu Dhabi 51900, United Arab Emirates; Fungus Testing Laboratory & Molecular Diagnostics Laboratory, Department of Pathology and Laboratory Medicine, University of Texas Health Science Center, San Antonio 78229, Texas, USA; Fungus Testing Laboratory & Molecular Diagnostics Laboratory, Department of Pathology and Laboratory Medicine, University of Texas Health Science Center, San Antonio 78229, Texas, USA; Department of Pediatrics and Department of Medical Microbiology and Immunology, College of Medicine and Health Sciences, Al Ain, Abu Dhabi 15551, United Arab Emirates; Zayed Bin Sultan Centre for Health Sciences, United Arab Emirates University, Al Ain, Abu Dhabi 15551, United Arab Emirates

**Keywords:** *Microsporum canis* complex, pediatric dermatophytosis, antifungal susceptibility, ITS sequencing, molecular epidemiology, UAE

## Abstract

*Microsporum canis* complex infections remain a major cause of pediatric dermatophytosis, yet molecular epidemiologic and antifungal susceptibility data from the Arabian Gulf are scarce, with no contemporary regional assessment since 1981–1988. We characterized the clinical, molecular, and antifungal susceptibility profiles of *M. canis* complex isolates in Abu Dhabi and assessed concordance between phenotypic and ITS-based identification. Fifty-two clinical dermatophyte isolates collected through passive surveillance at the UAEU Fungal Reference Laboratory (September 2024–December 2025) underwent phenotypic identification, ITS sequencing, and antifungal susceptibility testing (CLSI M38). Clinical data were available for 48 patients. ITS sequencing identified 47 isolates (90.4%) as *M. canis* and 5 (9.6%) as the *M. audouinii* clade, with phenotypic discordance in 9.6% of isolates (including one *M. canis* misidentified as *Trichophyton rubrum*). The cohort was predominantly pediatric (79.2% ≤12 years; 54.2% female). Tinea capitis predominated (60.4%), followed by tinea corporis (20.8%) and multifocal disease (16.7%). Cat exposure was reported in 50% and infected household contact in 27.1%. All isolates showed low minimum inhibitory concentrations (MICs ; terbinafine MIC₅₀/₉₀ 0.015/0.03; itraconazole ≤ 0.03/0.06; voriconazole ≤ 0.03/≤0.03; griseofulvin 0.125/0.25; fluconazole 4/8 µg/ml). Despite this, 81.2% (26/32) of patients with follow-up experienced treatment failure or recurrence, with no clear MIC–outcome association. This first molecular and antifungal characterization of *M. canis* complex infections in the UAE over three decades showed that ITS sequencing corrected phenotypic identification in nearly 10% of cases. High recurrence despite low MICs suggests limited predictive value of in vitro susceptibility, supporting species-directed management, molecular confirmation, species-directed management, and One Health strategies.

## Introduction

*Microsporum canis* belongs to the *M. canis complex* and forms a phylogenetically coherent lineage with the closely related anthropophilic species *M. audouinii* and *M. ferrugineum*, as demonstrated by multilocus analyses.^1^ These species are significant causes of pediatric dermatophytosis, particularly tinea capitis and tinea corporis. Despite historical separation based on host preference and macroconidial morphology, multilocus analyses reveal limited genetic divergence among these taxa, reflecting the broader ‘species problem’ in dermatophytes where genotypic similarity contrasts with distinct ecological and inflammatory profiles.^1^ This genotype-phenotype discordance suggests a continuum of host adaptation rather than discrete biological species.

*Microsporum canis* is a major zoophilic pathogen of feline and small animals, frequently implicated in human transmission clusters in household and school settings.^2,3^ Although predominantly zoophilic, it exhibits ecological plasticity with transmission occurring via animal, human, and environmental routes.^4,5^ Cats serve as the principal reservoir, with infection rates reaching up to 100% in some populations, while dogs act as secondary reservoirs (40%–90%).^6,7^ Human infection is acquired through direct or indirect contact with infected animals. Asymptomatic carriage in companion animals can facilitate household spread, as evidenced by frequent isolation from pets of patients with tinea corporis^8,9^ and documented intrafamilial zoonotic transmission.^10^ Importantly, infected cats may remain asymptomatic carriers, sustaining transmission and posing considerable challenges to infection control and eradication strategies.^11^ This reservoir dynamic is particularly relevant in the United Arab Emirates (UAE), where large companion and stray cat populations may enhance zoonotic exposure. Clinically, *M. canis* infections are typically inflammatory and may progress to kerion, a severe inflammatory form of tinea capitis characterized by a boggy, suppurative scalp lesion. *Microsporum canis–*associated tinea capitis remains a major superficial mycosis with rising global incidence,^12^ predominantly affecting pediatric populations, although infections also occur in adults.

The anthropophilic counterparts *M. audouinii* and *M. ferrugineum* primarily share close genetic relationships with *M. canis* yet exhibit overlapping phenotypic traits and variable conidiation complicating routine identification.^1,13^ Sequence-based confirmation is essential when accurate species delineation is clinically or epidemiologically important. Beyond taxonomic and diagnostic challenges, the clinical management of *M. canis* complex infections faces additional complexity. The lack of standardized clinical breakpoints for dermatophytes limits the ability to detect resistance and optimize therapeutic strategies.^14^

Historically, *M. canis* was identified as the predominant cause of tinea capitis in Al Ain, UAE, between 1981 and 1988.^15^ In the decades since, molecular epidemiologic and antifungal susceptibility data for the *M. canis* complex in the UAE and the broader Arabian Gulf region have been lacking. This prolonged gap is particularly significant given the predominance of pediatric disease, sustained zoonotic exposure, and the recognized discordance between *in vitro* susceptibility and clinical outcomes. The reliability of conventional phenotypic identification in this region has not been systematically assessed, and the relationship between species-level classification, antifungal susceptibility, and clinical outcomes remains undefined.

To address this gap, we conducted an integrated clinical, phenotypic, molecular, and antifungal susceptibility analysis of isolates in the *M. canis* species complex in Abu Dhabi. We aimed to confirm species identification, assess concordance between diagnostic methods, characterize susceptibility patterns, and evaluate associations with clinical outcomes, providing baseline data and highlighting key diagnostic and therapeutic challenges.

## Material and Methods

### Isolate Collection and Clinical Data

A collection of 52 clinical dermatophyte isolates recovered through routine passive surveillance from patients across Abu Dhabi, UAE, between September 2024 and December 2025 was investigated. Isolates were predominantly from tinea capitis and tinea corporis cases and were initially identified as belonging to the *M. canis* species complex based on macro- and micromorphology, supplemented by MALDI-TOF MS (bioMérieux, France). The collection included two isolates with no initial identification, one preliminarily reported as *Trichophyton rubrum*, and one as *M. audouinii*. All isolates were subcultured on Sabouraud dextrose agar with chloramphenicol (SDA-C) to ensure purity prior to detailed phenotypic and molecular analyses.

Anonymized clinical data, including demographic characteristics, travel history, animal exposure, comorbidities, immunosuppression status, clinical presentation, treatment regimens, and outcomes, were analyzed to evaluate associations between molecular genotypes within the *M. canis* species complex, antifungal susceptibility profiles, and clinical outcomes.

### Morphological and Physiological Characterization

All isolates (*n* = 52) were screened for purity using Lactophenol Cotton Blue (LPCB) tease mounts and subcultured on SDA-C (at 28°C for up to 15 days). After 1 week, a subculture was made on Potato Dextrose Agar (PDA) for slide culture and morphological assessment, including evaluation of colony texture and reverse pigmentation (ochraceous yellow or diffusible pigment production). Mature (>2-week-old) pure cultures were subjected to physiological testing, including hair perforation, rice grain, and urease tests. For the hair perforation assay, sterilized 1-cm fragments of pre-pubescent human hair were incubated with fungal inoculum in sterile distilled water at room temperature and examined weekly for up to 4 weeks for wedge-shaped perforations; *T. rubrum* served as a negative control.^16^ The rice grain test was performed using autoclaved polished rice inoculated with 14-day-old cultures and incubated at room temperature (∼25°C) for up to 14 days to assess growth, pigment production, and sporulation consistent with *M. canis* species complex.^17–19^ Urease activity was evaluated on Christensen’s urea agar incubated at 28°C and interpreted according to established criteria.^17^ All isolates were cryopreserved in 15% glycerol at –80°C and stored in sterile water at room temperature.

### Molecular Characterization

Genomic DNA was extracted from all isolates (*n* = 52) using the Fungi/Yeast Genomic DNA Isolation Kit (Norgen Biotek Corp., Thorold, ON, Canada) according to the manufacturer’s instructions. The complete internal transcribed spacer region (ITS1–5.8S–ITS2)was amplified using fungal primers BMB-CRF (5′-GTACACACCGCCCGTCG-3′) and ITS4R (5′- TCCTCCGCTTATTGATATGC -3′).^20^ polymerase chain reaction (PCR) amplification was performed as described by Cañete-Gibas et al. (2023).^21^ PCR products were purified using the Wizard SV Gel and PCR Clean-Up System (Promega, Madison, WI, USA) and sequenced bidirectionally using the BigDye Terminator v3.1 Cycle Sequencing Kit on SeqStudio Genetic Analyzer (Applied Biosystems, Thermo Fisher Scientific, Waltham, MA, USA). Sequences were edited and assembled using MEGA X v10.2.6 and BioEdit v7.7.1.^22,23^

All ITS sequences were subjected to an initial BLASTn search for species-level confirmation (https://blast.ncbi.nlm.nih.gov/Blast.cgi, accessed on 12-Jan-2026. As *M. canis* forms a species complex, BLASTn alone was insufficient for reliable discrimination; species identity was therefore confirmed by maximum-likelihood phylogenetic analysis against ex-type and authentic reference strains from the Westerdijk Fungal Biodiversity Institute/CBS collection retrieved from GenBank, anchoring assignments to verified type material. Reference strains comprised the ex-type or authentic reference strains of all accepted members of the *M. canis* complex—*M. canis* (CBS 496.86^T^), *M. audouinii* (CBS 545.93^T^), and *M. ferrugineum* (CBS 497.48)—alongside the closely related *M. distortum* (ATCC 26224) and additional reference sequences retrieved from GenBank, with *T. rubrum* (CBS 392.58) as the outgroup. Sequence alignments were generated using MUSCLE in MEGA X v10.2.6, visually inspected, and trimmed at both ends to a common length defined by the reference strains to remove ragged terminal overhangs. As the resulting ITS alignment was unambiguous across the complex, no internal positions were excluded, and all aligned sites were used for tree construction. The best-fitting nucleotide substitution model was determined in MEGA X using the Find Best DNA Models tool, based on the corrected Akaike Information Criterion (AICc). The Tamura-Nei model incorporating among-site rate variation (gamma distribution with a proportion of invariant sites; TN93 + G + I) was among the best-fitting models by AICc and was applied for maximum-likelihood inference. A maximum-likelihood tree was inferred under the Tamura-Nei model with gamma-distributed rates and a proportion of invariant sites (TN93 + G + I), using the Nearest-Neighbor-Interchange heuristic with a Neighbor-Joining/BioNJ starting tree. Node support was assessed with 1000 bootstrap replicates, and the tree was rooted using *Trichophyton rubrum* (CBS 392.58) as the outgroup. The final tree was visualized in FigTree v1.4.4 (http://tree.bio.ed.ac.uk/software/figtree/), accessed on 30-Jan-2026 and iTOL v7.^24^

### Antifungal Susceptibility Testing

*In vitro* antifungal susceptibility testing was performed on a subset of 29 viable isolates within the *M. canis* complex (27 *M. canis* and 2 *M. audouinii*) using the Clinical and Laboratory Standards Institute (CLSI) M38 broth microdilution method at the Fungus Testing Laboratory, Department of Pathology and Laboratory Medicine, University of Texas Health Science Center at San Antonio, TX, USA. Antifungal susceptibility testing was not available in-house and was therefore performed externally. Due to logistical and financial constraints, testing was limited to 29 available viable isolates, which were selected based on availability rather than phenotype, morphology, or suspected antifungal resistance and represented the geographic and morphological diversity of the study collection. *In vitro* activity was assessed for five antifungal drugs: fluconazole, itraconazole, voriconazole, terbinafine, and griseofulvin. Stock solutions were prepared in dimethyl sulfoxide (DMSO) at 100X the final testing concentration, with subsequent dilutions made in RPMI1640 medium buffered to pH 7.0 using 0.165 M MOPS (Sigma-Aldrich, Germany). The final testing concentrations were terbinafine 0.004–2 μg/ml; fluconazole 0.125–64 μg/ml; and itraconazole, voriconazole, and griseofulvin 0.03–16 μg/ml. The quality control strain used was *Trichophyton mentagrophytes* MRL 1957 (ATCC MYA-4439). The final DMSO concentration in the test assay was 1% v/v.

### Statistical Analysis

Statistical analyses were performed in Python 3.12 with SciPy 1.11. Categorical data are summarized as frequencies and percentages, continuous non-minimum inhibitory concentration (MIC) variables as medians and ranges, and MICs as geometric means (GMs) on the log₂ dilution scale. Categorical variables were compared using Fisher’s exact or chi-square tests; MIC distributions between outcome groups were compared by Mann–Whitney U test and by Welch’s *t*-test on log₂-transformed values. Significance was defined as *P* < 0.05 (two-tailed); findings were interpreted cautiously given small subgroup sizes and the ±1 log₂ essential-agreement variability of broth microdilution.

## Results

### Clinical Characteristics and Treatment Outcomes

Complete clinical data were available for 48 patients (92.3%), and subsequent clinical analyses were conducted on this subset.

### Demographic and Epidemiological Characteristics

**Figure 1.**
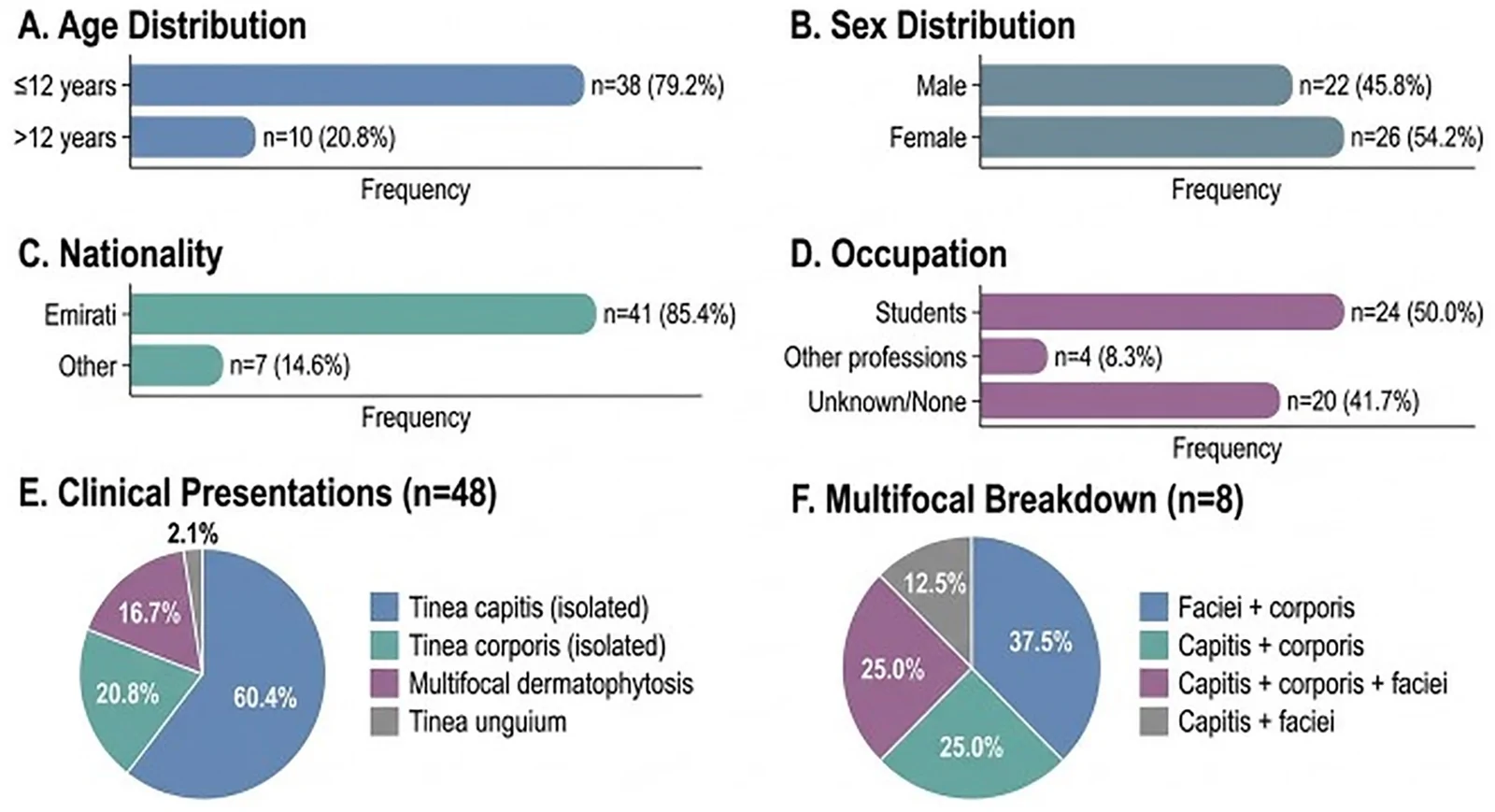
Demographic, epidemiological, and clinical characteristics of patients with *M. canis* complex infections in the United Arab Emirates (*n* = 48). (A) Age distribution demonstrating predominant pediatric presentation (79.2% aged ≤ 12 years). (B) Sex distribution showing female predominance (54.2%). (C) Nationality composition with majority of Emirati nationals (85.4%). (D) Occupational distribution showing students as the largest group (50.0%). (E) Proportional distribution of clinical presentations, with tinea capitis as the most common manifestation (60.4%, *n* = 29), followed by tinea corporis (20.8%, *n* = 10), multifocal dermatophytosis (16.7%, *n* = 8), and tinea unguium (2.1%, *n* = 1). (F) Breakdown of multifocal dermatophytosis subtypes (*n* = 8), showing concurrent faciei and corporis infections as the predominant pattern (50.0%, *n* = 4), followed by capitis and corporis (25.0%, *n* = 2), and capitis, corporis, and faciei (25.0%, *n* = 2).

The study cohort (*n* = 48; age range 7 months–41 years) was predominantly pediatric (≤12 years; 79.2%, 38/48), with a slight female predominance (54.2%) and a majority of Emirati nationals (85.4%). Occupational and travel histories were frequently undocumented (unavailable in 41.7% and 87.5%, respectively); one patient reported international travel to Thailand for medical purposes. The full demographic and epidemiological breakdown is shown in Figure 1A–D.

### Clinical Manifestations

Multifocal dermatophytosis was documented in 16.7% (8/48) of cases, involving various combinations of tinea capitis, corporis, and faciei (subtype breakdown in Fig. 1F).

### Underlying Comorbidities and Immunocompromised Status

Immunocompromising conditions were identified in 4.2% (2/48) of patients, including one receiving active treatment for dermatomyositis and one with acute lymphoblastic leukemia (ALL).

Comorbidities were identified in 35.4% (17/48) of the patients. The reported conditions included obesity, alopecia areata, atopic dermatitis, cataract, amblyopia, asthma, dyslipidemia, dermatomyositis, G6PD deficiency, hypertension, hypospadias, nephrectomy, polycystic ovary syndrome (PCOS), anemia, prediabetes, and T- cell acute lymphoblastic leukemia. Among the comorbidities recorded in this cohort, atopic dermatitis was the most commonly reported (29.4%, 5/17).

### Zoonotic Exposure and Transmission

**Animal Contact:** Cat exposure was most frequently reported (50%, 24/48), including one case with microbiologically confirmed feline dermatophytosis. Combined exposure to cats with birds and cats with rabbits was reported in 4.2% (2/48) each. Exposure to birds alone was documented in 2.1% (1/48), while contact with unspecified pets was reported in 10.4% (5/48). Animal exposure history was unknown in 29.2% (14/48).

**Human-to-human transmission:** Contact with an infected household member was reported in 13 cases (27.1%,13/48), and exposure to an infected schoolmate in one case (2.1%,1/48). Five patients (10.4% 5/48) reported no known contact with infected individuals. However, contact history was unavailable for the majority of the cohort 60.4% (29/48).

### Treatment and Clinical Outcomes

Treatment data were available for 97.9% of patients (47/48). Topical monotherapy was prescribed in 25.5% (12/47), whereas combination therapy with systemic and topical agents was used in 74.5% (35/47).

Among patients receiving systemic antifungal therapy as part of their initial regimen (*n* = 35), terbinafine was the most frequently used agent (57.1%, 20/35), followed by fluconazole (28.6%, 10/35) and itraconazole (11.4%, 4/35). One patient received concomitant terbinafine and fluconazole (2.9%, 1/35). Treatment regimens and detailed responses are summarized in Supplementary Table S1.

Follow-up data were available for 66.7% patients (32/48). For the purposes of this retrospective study, and in view of the absence of universally standardized definitions for these clinical outcome terms,^25^ clinical outcomes were categorized as treatment failure, recurrence, or sustained clinical improvement. Treatment failure referred to the absence of clinical improvement following one treatment cycle, whereas recurrence referred to persistent or worsening infection requiring additional treatment after completion of the initial treatment cycle. Clinical outcomes were assessed after completion of therapy, consistent with the outcome reporting approach adopted by Vanam et al., in which patients were categorized according to clinical improvement or recurrence following therapy.^26^ Among these, 81.2% (26/32) experienced treatment failure or recurrence after their first cycle of therapy, while 18.8% (6/32) achieved sustained clinical improvement. No significant associations were observed between outcome and age group (pediatric vs. adult), sex, or clinical presentation. However, treatment modality showed a significant association (*P* = 0.0017) with clinical outcome. Patients receiving topical therapy alone had better outcomes (100% improvement, 4/4) compared to those receiving combination systemic and topical therapy (7.1% improvement, 2/28). This likely reflects differences in initial disease severity, with combination therapy preferentially used in more severe or refractory cases. Among patients with documented outcomes, *M. canis* predominated (93.8%, 30/32), with only 2 *M. audouinii* infections identified. Both *M. audouinii* cases experienced recurrence; however, the small number of cases precluded meaningful statistical comparison of species-specific treatment outcomes.

Among patients who experienced recurrence (*n* = 26): treatment failure occurred after the first cycle of systemic antifungal therapy in 11.5% (3/26) and after the first cycle of topical monotherapy in 38.5% (10/26), suggesting that topical therapy alone may be insufficient in some cases. However, 50% (13/26) also experienced recurrence despite receiving first cycle of combination systemic and topical therapy, indicating that factors beyond treatment modality may contribute to treatment failure.

### Phenotypic and Adjunct Physiological Characterization of *M. audouinii* and *M. canis* Isolates

**Figure 2.**
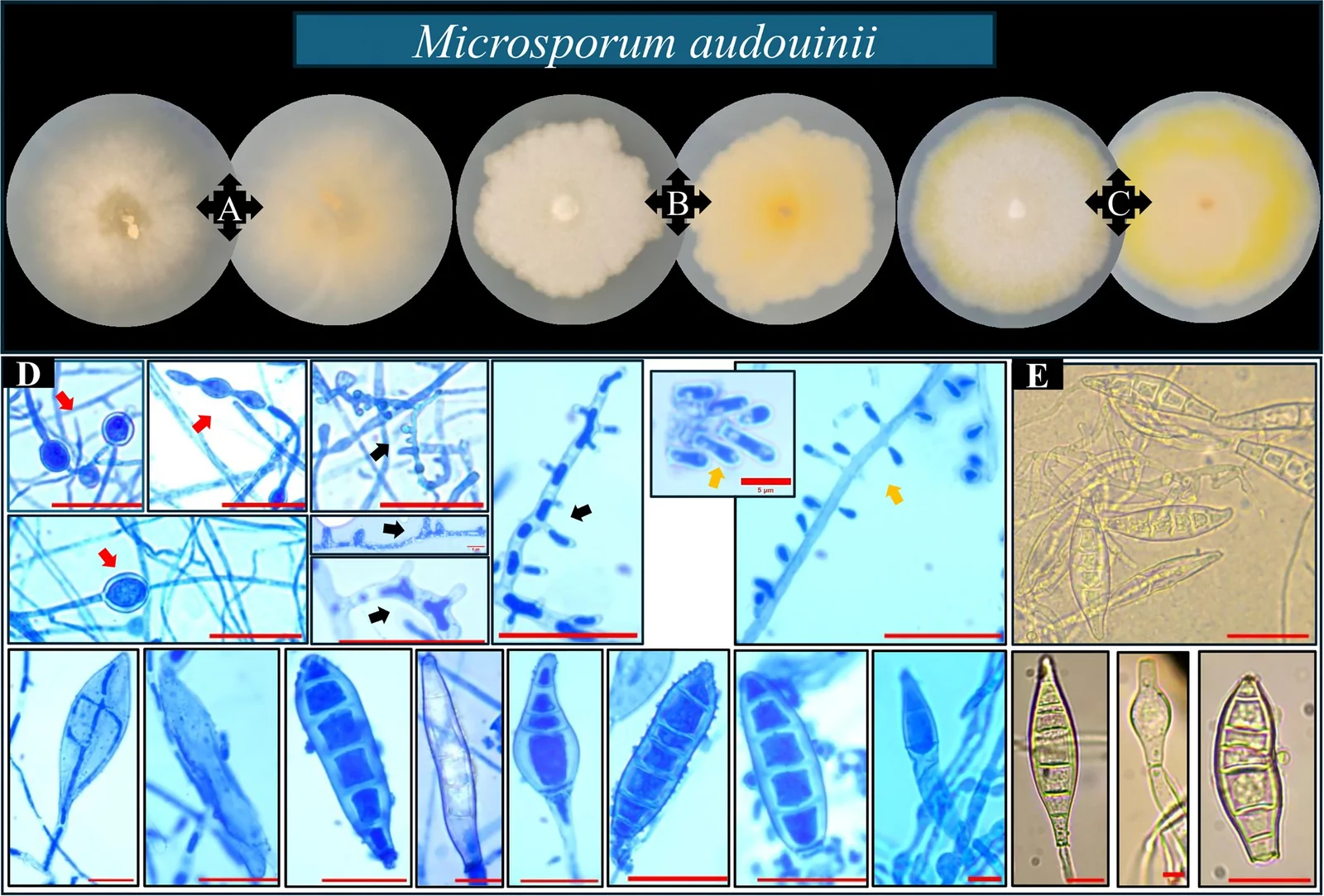
Colony morphology of *Microsporum audouinii* isolates on PDA after 14 days of incubation.(A) Classical *M. audouinii* phenotype. (B–C) Divergent *M. audouinii* colony morphotypes characterized by increased aerial mycelium and enhanced conidiation. (D) Microscopic morphology showing terminal and intercalary chlamydospores **(red arrows)**, pectinate hyphae **(black arrows)**, clavate to ovoid microconidia **(yellow arrows)**, and macroconidia illustrating morphological variation **(lower panels)**. (E) Macroconidia in 10% lactic acid mount preparations illustrating macroconidial morphology and septation. Scale bar = 20 µm.

Five *M. audouinii* isolates were examined. After 14 days of incubation on PDA, the isolates segregated into three distinct morphotypes (Fig. 2A–C). One isolate exhibited the classical *M. audouinii* phenotype, forming flat, spreading colonies with sparse aerial mycelium and a thin, suede-like surface. The obverse was greyish- to tannish-white, while the reverse showed distinct central rose-brown pigmentation (Fig. 2A). Conidiation was limited, and microscopic examination revealed numerous terminal and intercalary chlamydospores, with sparse macro- and microconidia (Fig. 2D–E).

In contrast, the remaining isolates displayed divergent *M. audouinii* colony morphotypes characterized by increased aerial mycelium and enhanced conidiation (Fig. 2B–C). Colony texture ranged from flat to granular or coarsely fluffy, and one morphotype exhibited peripheral yellow pigmentation with partial resemblance to *M. canis*. Microscopic examination demonstrated abundant macroconidia, many with irregular morphology, whereas chlamydospores were less prominent than in the classical phenotype (Fig. 2D–E).

**Figure 3.**
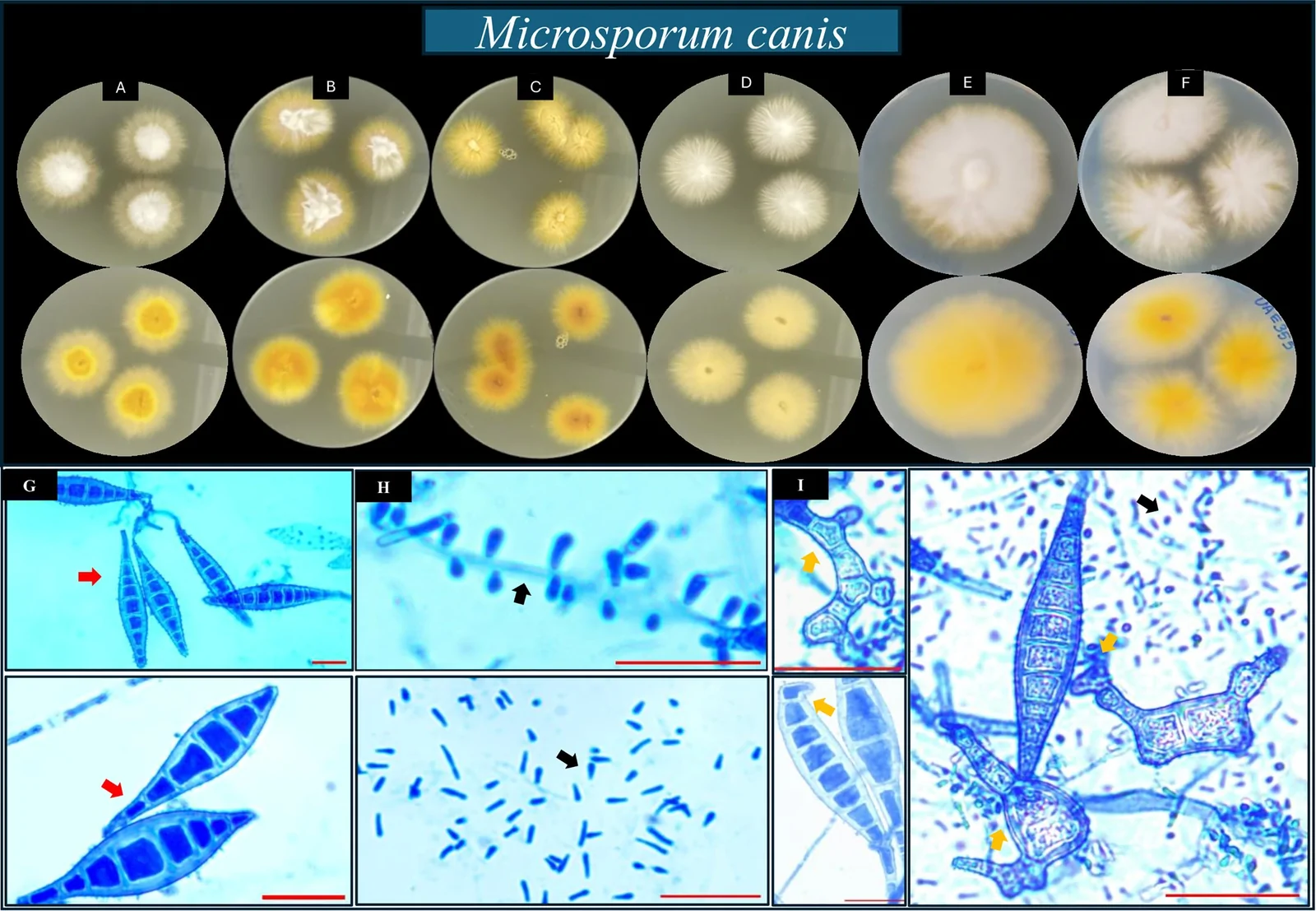
Macroscopic and microscopic morphology of *M. canis* on PDA after 14 days of incubation. (A–F) Obverse and reverse colony morphologies demonstrating phenotypic variability. (G) Characteristic macroconidia, spindle-shaped with rostrate or beak-like apical cells, basal frills, and lateral wall projections (**red arrows**). (H) Clavate to pyriform microconidia (**black arrows**). (I) Representative atypical microscopic features, including *M. canis* distortum-like morphology (UAE355) with contorted macroconidia (**yellow arrows**) and distinctly club-shaped microconidia (**black arrows**). Scale bar = 20 µm.

Forty-seven *M. canis* isolates on PDA (Fig. 3A–F) demonstrated marked macromorphological heterogeneity, with colony texture ranging from thin to densely woolly and reverse pigmentation varying from intense ochraceous-yellow to faint or no pigment. Several isolates exhibited uniformly downy growth with reduced conidiation; one isolate (UAE268), received from a referral laboratory as *T. rubrum*, remained predominantly sterile on primary culture and developed characteristic *M. canis* macroconidia only after repeated subculture from localized conidiating sectors. Microscopic examination revealed abundant, thick-walled macroconidia in most isolates (Fig. 3G–I), with variation in septation, size, and morphology, including occasional irregular or dysgonic forms. Representative microconidia and atypical microscopic features, including the *M. canis* distortum-like variant, are also shown (Fig. 3H–I). A comprehensive summary of morphological characteristics for all isolates is provided in Supplementary Table S2.

### Adjunct Physiological Testing

**Figure 4.**
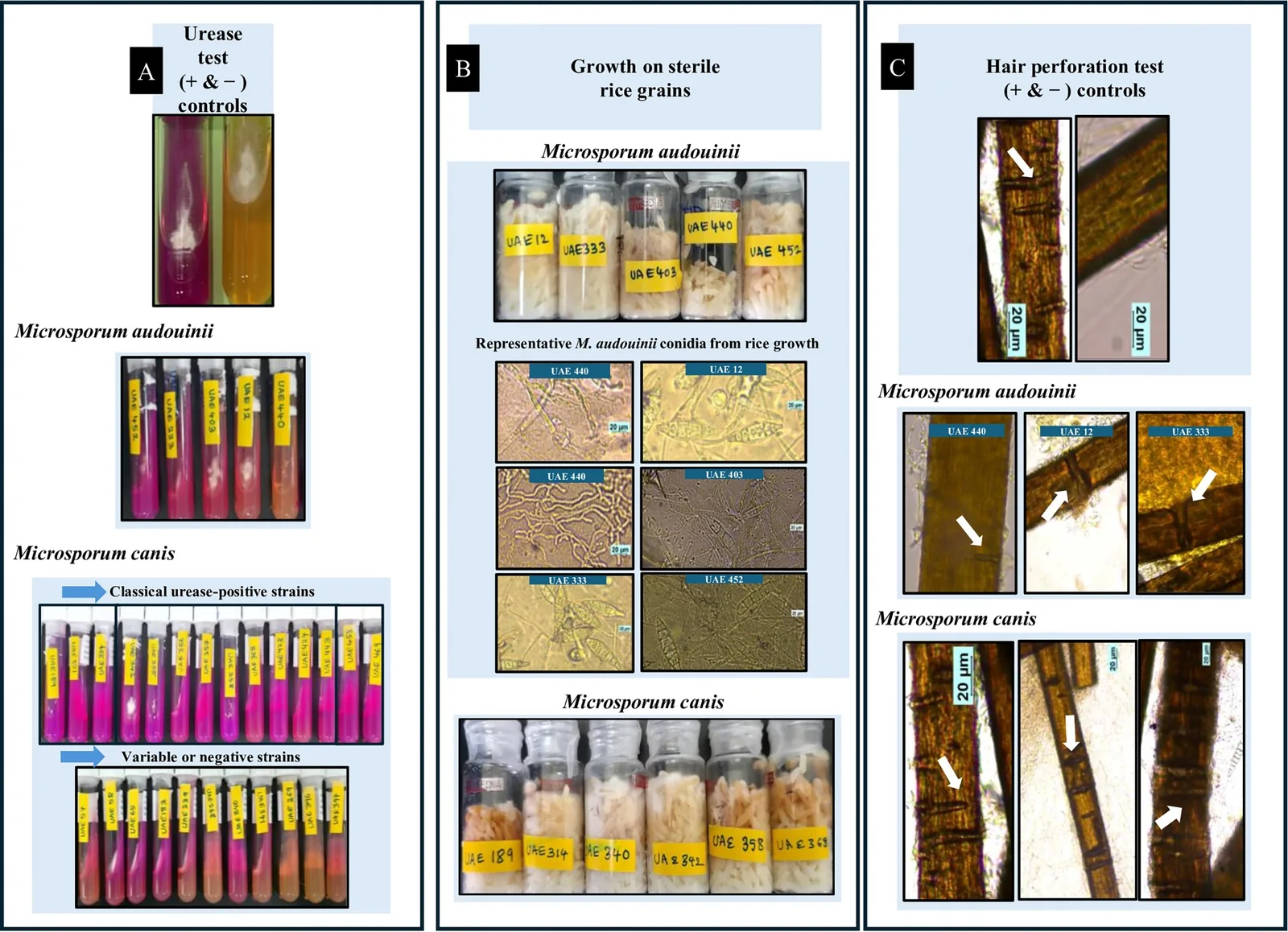
Adjunct physiological characteristics of *M. audouinii* and *M. canis*. (A) Urease test (7–10 days) showing positive (+), weak (±), and negative (−) reactions with positive and negative controls. (B) Growth, pigment production, and representative conidiation on sterile rice grains in *M. audouinii* including the classical phenotype and divergent colony morphotypes. (C) Hair perforation test (≥14 days) demonstrating keratinolytic activity with representative perforation patterns in both groups (arrows). Scale bars = 20 µm.

**Figure 5.**
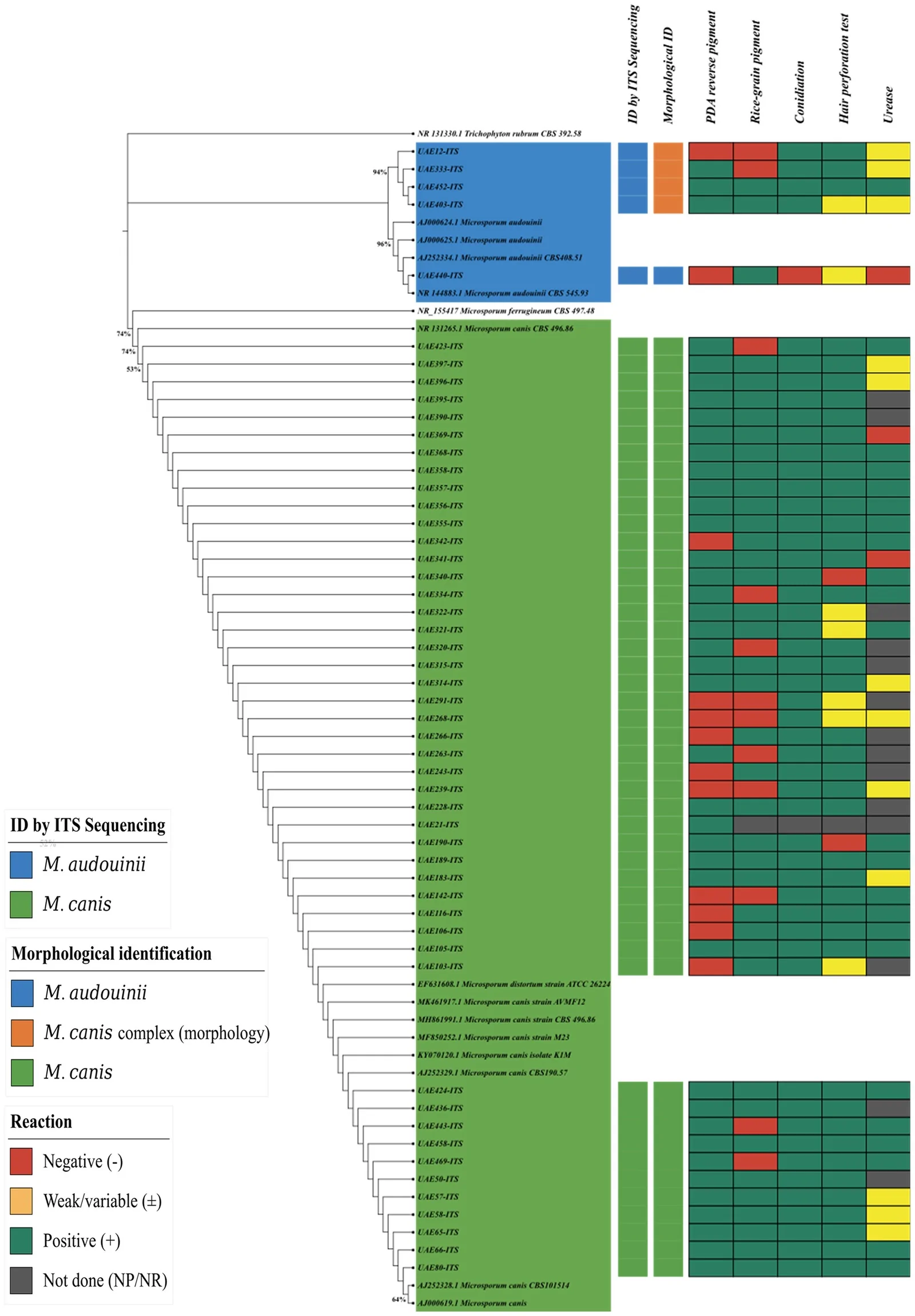
Maximum likelihood tree constructed from ITS sequences showing the relationship between 52 clinical isolates and reference strains. *Trichophyton rubrum* CBS 392.58 was used as the outgroup. Bootstrap values are displayed at the nodes. Branch lengths are proportional to genetic distance. Two adjacent strips show identification by ITS sequencing and morphological identification (*M. audouinii*; *M. canis*; *M. canis* complex by morphology). The outer heatmap shows five adjunct phenotypic tests (PDA reverse pigment, rice-grain pigment, conidiation hair perforation test, urease), scored as negative (–), weak/variable (±), positive (+), or not done/not revived. Reference strains are left blank in the metadata panels.

Adjunct physiological testing was performed on 51 isolates within the *M. canis* species complex (Fig. 4A–C; Fig. 5; Supplementary Table S2). Urease testing of the five *M. audouinii* isolates showed variable enzymatic activity. Three of the four isolates exhibiting divergent colony morphotypes were weakly or strongly urease positive, whereas the isolate displaying the classical *M. audouinii* phenotype was urease negative. In contrast, urease activity among the *M. canis* isolates was also variable, with 33 of 47 isolates (70.2%) demonstrating positive or weak urease activity, while the remainder were negative.

Pigment production and conidiation on sterile rice grains were likewise variable (Fig. 4B). Most isolates produced yellow to brownish pigmentation accompanied by conidial growth, whereas a minority remained non-pigmented. The *M. audouinii* isolates demonstrated characteristic macroconidial development on rice substrates, while *M. canis* isolates produced macroconidia irrespective of pigment production.

Hair perforation testing revealed keratinolytic activity in the majority of isolates (Fig. 4C). Three of the four isolates with divergent *M. audouinii* morphotypes were positive, whereas one isolate (UAE403) and the isolate displaying the classical *M. audouinii* phenotype (UAE440) exhibited weak or borderline reactions characterized by limited focal perforations. Among *M. canis*, hair perforation was positive in most isolates, although six isolates demonstrated weak or absent keratinolytic activity. Positive and negative controls confirmed assay validity. Detailed strain-wise physiological results are summarized in Fig. 4 and Supplementary Table S2.

Overall, adjunct physiological characteristics demonstrated notable intra-species variability and overlapping profiles between *M. audouinii* and *M. canis*.

### Phylogenetic Analysis and Species Discrimination

Phylogenetic analysis of ITS sequences revealed distinct clustering patterns within the *M. canis* species complex (Fig. 5). Of the 52 isolates analyzed, 47 (90.4%) clustered with *M. canis* type strain CBS 496.86T and other reference strains showing 100% ITS sequence identity, confirming their identification as *M. canis*. Among the 47 *M. canis* isolates, 3 exhibited poor or no conidiation at the time of initial examination, leading to preliminary misidentification (i.e., 2 had been submitted without species identification, and 1 had been incorrectly submitted as *T. rubrum*). In isolate UAE268, characteristic *M. canis* macroconidia and sparse microconidia became evident only after repeated subcultures, with conidiation restricted to localized sectors of the colony. The sparse and delayed sporulation observed in these isolates likely contributed to the initial identification challenges and highlights the limitations of morphology-based identification when conidial production is suboptimal.

The remaining five isolates (9.6%) clustered distinctly with the *M. audouinii* type strain CBS 545.93^T^ and other related strains, segregating clearly from the *M. canis* clade. Phylogenetic analysis confirmed these as *M. audouinii*, correcting initial misidentification in four cases that had been preliminarily reported as *M. canis* by the referring laboratory, while the fifth isolate (UAE440) had been correctly identified as *M. audouinii*. All ITS sequences generated in this study have been deposited in the NCBI GenBank database under accession numbers (GenBank accession: PZ274327-PZ274378).

### Correlation of ITS Phylogeny with Morphological and Physiological Characteristics

Integration of the ITS phylogeny with the morphological and physiological characteristics of all study isolates (Fig. 5; Supplementary Table S2) showed that phenotypic diversity was not consistently associated with ITS sequence variation. Within the *M. audouinii* clade, the classical isolate exhibited a phenotypic profile distinct from the ITS genotype variants despite their close phylogenetic relationship. Likewise, *M. canis* isolates, which shared 100% ITS sequence identity, displayed considerable variation in colony morphology, pigmentation, conidiation, urease activity, and hair perforation. Collectively, these findings demonstrate that while ITS sequencing reliably resolved species-level identification, it did not capture the intraspecific phenotypic diversity observed within the *M. canis* complex.

### Antifungal Susceptibility Testing

Broth microdilution testing was performed on 29 *M. canis* complex isolates. Terbinafine, itraconazole, and voriconazole demonstrated particularly low MIC, whereas fluconazole exhibited comparatively higher values. MIC₅₀/MIC₉₀ values were as follows: fluconazole, 4/8 µg/ml; itraconazole, ≤0.03/0.06 µg/ml; voriconazole, ≤0.03/≤0.03 µg/ml; terbinafine, 0.015/0.03 µg/ml; and griseofulvin, 0.125/0.25 µg/ml. Detailed MIC ranges are presented in Table 1.

**Table 1.** *In vitro* antifungal susceptibility profiles of *M. canis* species complex isolates (*n* = 29).

Antifungal	MIC Range (µg/ml)	MIC₅₀ (µg/ml)	MIC₉₀ (µg/ml)
Fluconazole	0.5–32	4	8
Itraconazole	≤0.03–0.125	≤0.03	0.06
Voriconazole	≤0.03–0.06	≤0.03	≤0.03
Terbinafine	≤0.004–0.06	0.015	0.03
Griseofulvin	≤0.03–0.25	0.125	0.25

Despite phylogenetic divergence, *M. canis* and *M. audouinii* isolates demonstrated comparable in vitro antifungal susceptibility profiles, with median MIC values differing by less than twofold across all tested agents.

Among patients with both antifungal susceptibility testing (AFST) results and outcome data (*n* = 18), 5 cases (27.8%) demonstrated clinical improvement and 13 cases (72.2%) experienced recurrence. MIC values showed no significant association with treatment outcome for any of the five antifungals tested. GM terbinafine MICs were comparable between groups (improvement 0.015 µg/ml vs. recurrence 0.015 µg/ml; Mann–Whitney *P* = 0.96), as were GM MICs for fluconazole (4.00 vs. 3.06 µg/ml; *P* = 0.42), itraconazole (0.053 vs. 0.033 µg/ml; *P* = 0.059), voriconazole (0.030 vs. 0.033 µg/ml; *P* = 0.42), and griseofulvin (0.061 vs. 0.060 µg/ml; *P* = 1.00). A parametric comparison of geometric means on log₂-transformed MICs likewise showed no significant difference for any agent (all *P* > 0.15).

## Discussion

This study provides the first molecular characterization of the *M. canis* species complex among clinical dermatophytosis cases in the UAE, in a cohort dominated by pediatric tinea capitis. The predominance of *M. canis* and its strong association with childhood scalp infection are consistent with the zoonotic, cat-associated epidemiology of this species.

ITS sequencing resolved phenotypic misidentification in 9.6% of cases (5/52), revealing misidentifications at both the species level and genus level. Four isolates initially classified as *M. canis* based on conventional morphological criteria were subsequently reclassified as *M. audouinii* following phylogenetic analysis, while one isolate provisionally identified as *T. rubrum* was confirmed as *M. canis*. These discrepancies highlight limitations of morphology-based identification, particularly when conidiation is atypical, with misclassification occurring both within the *M. canis* complex and across dermatophyte genera. Notably, intergeneric misidentification of *T. rubrum* as *M. canis* underscores that even established diagnostic features can be misleading under atypical phenotypic conditions.

Notably, European *M. audouinii* isolates exhibiting *M. canis—*like macroconidia, rice growth, and hair perforation were confirmed as *M. audouinii* by ITS sequencing (99.9% homology),^27^ illustrating genotype—phenotype discordance within conserved lineages. This is consistent with recent phylogenetic syntheses showing that the *M. canis* clade comprises four closely related evolutionary lineages: the zoophilic *M. canis*, the anthropophilic *M. audouinii* and *M. ferrugineum*, and a MAT1-2 lineage represented by the historical *Nannizzia otae* mating tester. The close phylogenetic affinity of the MAT1-2 lineage to both *M. canis* and *M. audouinii* further underscores the evolutionary complexity of this clade and highlights the limited resolving power of the ITS barcode for closely related taxa.^28^ Likewise, the low intraspecific genetic divergence in *M. canis* across regions and hosts^29^ contrasts with its phenotypic variability, indicating that morphological expression within this lineage is largely shaped by ecological and host-driven factors beyond ITS sequence variation. Consistent with these observations, the present study identified substantial morphological and physiological variation among isolates sharing identical or nearly identical ITS sequences, further supporting the limited ability of the ITS barcode to reflect intraspecific phenotypic diversity within the *M. canis* complex.

In our cohort, the five *M. audouinii* isolates segregated into three distinct morphotypes on PDA cultures, ranging from classical flat, suede-like colonies with rose-brown pigmentation (Morphotype A) to more *M. canis*-like granular colonies with yellow pigmentation and abundant macroconidia (Morphotypes B and C). Recent phylogenomic analysis of the *M. canis* species complex identified 12 distinct genotypes despite minimal genetic divergence, with significant phenotypic heterogeneity observed across strains that shared identical ITS sequences.^12^ This genotype-phenotype discordance reflects adaptive evolution within the *M. canis* complex and underscores the need for molecular confirmation, as species-level identification guides therapy and epidemiological tracking.

The predominance of pediatric cases is consistent with established epidemiological patterns and likely reflects increased animal contact and heightened susceptibility of the prepubertal scalp.^8,30^ A recent 12-year Israeli cohort study (2010–2021) demonstrated that *M. canis* infections were exclusively confined to patients under 16 years of age, with 96% occurring in children younger than 11 years.^31^ The predominance of tinea capitis reflects the characteristic age-related and anatomic predilection of *M. canis* infections. The slight female predominance (54.2%) observed in this study aligns with some regional reports,^32–34^ although the clinical significance of sex-based differences in dermatophytosis remains unclear.

The high frequency of reported animal exposure, particularly contact with cats (50%), reinforces the zoonotic nature of *M. canis* infection in this cohort.^6,7^ Age-specific patterns of animal contact have been well documented, with children demonstrating significantly higher exposure rates (55.29%) compared to adults (34.78%) in recent epidemiological studies,^35^ consistent with the observation in our cohort. This age-related risk likely reflects more frequent and intimate contact between children and companion animals, increasing exposure to zoophilic pathogens.^35^ In the UAE, large companion and stray cat populations likely sustain zoonotic transmission from asymptomatic carriers, contributing substantially to human *M. canis* infections and complicating disease control.

The discordance between in vitro antifungal susceptibility and clinical outcomes represents the most significant finding of this study and warrants careful interpretation. All 29 *M. canis* species complex isolates tested demonstrated low MIC values for terbinafine (MIC₉₀ 0.03 µg/ml), itraconazole (MIC₉₀ 0.06 µg/ml), voriconazole (MIC₉₀ ≤0.03 µg/ml), and griseofulvin (MIC₉₀ 0.25 µg/ml), consistent with wild-type susceptibility profiles reported in the recent 27-year Chinese multicenter study by Liang et al. (2025).^36^ The study established upper limits of wild-type minimum inhibitory concentration (UL-WT MIC) distributions and reported generally low MIC values overall, although elevated fluconazole MICs and sporadic non–wild-type (NWT) isolates for terbinafine and griseofulvin were observed.^36^ The first documented terbinafine-resistant *M. canis* feline isolate (MIC >32 µg/ml) further underscores the potential for resistance under therapeutic pressure.^37^ Although azoles and terbinafine remain largely effective *in vitro*, clinical response does not consistently correlate with MIC values.^36^ Despite these favorable in vitro profiles, 81.2% of patients (26/32) with available follow-up data experienced treatment failure or recurrence, including 72.2% (13/18) of those with paired susceptibility and outcome data. This recurrence rate is substantially higher than reported in a contemporaneous Israeli pediatric cohort,^29^ where *M. canis* demonstrated 66%–73% clinical cure rates with fluconazole and griseofulvin, respectively, despite similar low MIC profiles.

Several factors likely explain why favorable *in vitro* profiles did not translate into clinical cure. Pharmacokinetic considerations are particularly salient in our predominantly pediatric cohort: weight-based dosing variability, incomplete gastrointestinal absorption, and suboptimal drug penetration into the hair shaft—the principal site of infection in tinea capitis—may yield inadequate drug concentrations at the target site despite systemic drug levels sufficient to inhibit fungal growth in vitro.^38^ Antifungal accumulation in keratinized tissues requires extended treatment courses, and terbinafine and azoles show penetration kinetics poorly predicted by serum drug levels alone^39^; empirically, mycological cure in *M. canis* tinea capitis has required a median treatment duration of 10 weeks,^31^ far exceeding the timeframe of standard *in vitro* susceptibility testing. Consistent with this, no antifungal MIC differed significantly between the clinical-improvement and recurrence groups in our cohort, and Liang et al. (2025)^36^ similarly reported no correlation between MIC and outcome—reinforcing that conventional AFST has limited utility for predicting clinical response in dermatophyte infections.

Host- and exposure-related factors likely compounded this *in vitro*/*in vivo* discordance. Adherence is a recognized challenge in pediatric populations—palatability of oral formulations, prolonged courses, and inconsistent administration may all have produced subtherapeutic exposure—though adherence was not systematically captured in this retrospective cohort. Furthermore, true relapse could not be distinguished from reinfection without longitudinal molecular typing; given that 50% of patients reported cat exposure and 27.1% documented infected household contacts, some apparent ‘recurrences’ may represent new acquisitions from untreated animal or human reservoirs rather than antifungal failure per se. Collectively, these observations indicate that clinical outcome in this cohort was driven principally by host factors, disease severity, adherence, and environmental reinfection rather than by in vitro susceptibility.

Griseofulvin, the first-line therapy for *Microsporum* infections, was not utilized due to limited availability in the UAE, despite evidence of species-specific efficacy.^40,41^ Terbinafine generally performs better for *Trichophyton* infections, whereas griseofulvin and fluconazole demonstrate equivalent efficacy for *Microsporum* infections.^42^ For *M. canis*, species-directed therapy is critical. Despite excellent *in vitro* activity (MIC₉₀ 0.03 µg/ml), terbinafine achieved 0% clinical cure in tinea capitis (0/4; 75% no response), compared to 95% cure in *T. tonsurans* infections (19/20; *P* < 0.001).^31^ In contrast, griseofulvin and fluconazole showed comparable efficacy for *M. canis* (73% and 66% cure, respectively; *P* = 0.44), supporting guideline recommendations that terbinafine be reserved for *Trichophyton* species,^43^ while *M. canis* infections are best treated with griseofulvin or azoles.^31^

For tinea capitis, current European guidelines recommend combined systemic and adjunctive topical therapy, with terbinafine preferred for *Trichophyton* infections and griseofulvin or itraconazole for *Microsporum* infections.^44^ Our findings are consistent with this approach, with 74.5% of patients receiving combination therapy. However, the lower cure rate in our UAE cohort (18.2%) compared with the 66–73% reported in Israel for *M. canis*, despite similar in vitro susceptibility profiles, underscores that in vitro susceptibility alone may not reliably predict clinical outcomes.^31^ This observation is consistent with recent European guidance, which notes that favorable terbinafine *in vitro* activity does not necessarily translate into high clinical cure rates, particularly in *Microsporum* infections, suggesting that host- and treatment-related factors may influence therapeutic outcomes beyond intrinsic antifungal susceptibility.^44^

The apparent superiority of topical monotherapy (100% improvement vs. 7.1% with combination therapy) likely reflects confounding by indication, as combination therapy was preferentially used for more severe or extensive disease, while topical therapy was reserved for mild, localized cases. Thus, the observed association reflects baseline disease severity rather than true treatment efficacy. Notably, recurrence remained high even with combination therapy (50%), indicating that treatment failure is multifactorial. Contributing factors likely include inadequate treatment duration, suboptimal adherence in pediatric populations, and ongoing zoonotic or household re-exposure, with some cases representing reinfection rather than true relapse. The high recurrence following topical therapy (38.5%) further underscores its limitations in tinea capitis, where systemic therapy is required to achieve effective drug penetration into hair shafts and follicles.

### Limitations

This study has several limitations. The overall cohort was relatively small (*n* = 52 isolates), and antifungal susceptibility testing was performed on a subset of isolates (the 29 *M. canis* complex isolates), while the subset with paired susceptibility and outcome data was smaller still (*n* = 18), reducing statistical power. Its retrospective design limited assessment of treatment adherence and duration—key determinants of therapeutic success, particularly in pediatric populations—and follow-up data were incomplete (66.7%). Although isolates were drawn from multiple centers, all were located within Abu Dhabi and collected through passive surveillance, so findings may not be generalizable to the wider UAE or region. The inability to distinguish relapse from reinfection, given high rates of animal and household exposure, further limits interpretation, as does the non-use of griseofulvin owing to limited regional availability. Finally, although ITS is the accepted primary barcode for dermatophytes and reliably resolved species-level identity, its discriminatory power is limited. Multilocus analysis using additional markers such as β-tubulin (BenA) or translation elongation factor 1-α (TEF1-α), or whole-genome/phylogenomic approaches, would provide greater intraspecific resolution and assessment of strain-level variation and virulence factors, and should be incorporated in future work.

## Conclusion

This study provides the first molecular and antifungal susceptibility characterization of *M. canis* complex infections in the UAE in over three decades, demonstrating diagnostic limitations and a clear discordance between in vitro susceptibility and clinical outcomes, indicating that MIC values do not predict treatment response.

These findings support species-directed management guided by infection site and treatment duration rather than susceptibility data alone. The pediatric predominance, zoonotic transmission, and high recurrence rates highlight the need for integrated One Health approaches. Limited access to griseofulvin represents a key gap in regional care. Prospective studies incorporating standardized treatment, adherence monitoring, and molecular approaches are needed to optimize management and inform regional guidelines.

## Supplementary Material

myag091_Supplemental_Files

## Data Availability

All ITS sequences generated in this study have been deposited in the NCBI GenBank database with accession numbers PZ274327-PZ27437. The data that support the findings of this study are available on request from the corresponding author.
