# Supplementary material for: Microsporum canis complex infections in the United Arab Emirates: Clinical, molecular, and antifungal susceptibility profiles
Source: Med Mycol. 2026 Aug 29;64(9):myag091. doi: 10.1093/mmy/myag091 (PMC13570593; doi:10.1093/mmy/myag091)
Supplement: Supplementary file 1 [file myag091_supplemental_files.zip › mm-2026-0224-File008.docx]

**Supplementary Table S1. Treatment regimens and detailed clinical responses.**

*Antifungal regimens by treatment cycle for clinical Microsporum canis complex isolates (n = 48). Em dash (—) denotes no further treatment cycle recorded.*

| **Strain no.** | **First cycle** | **Second cycle** | **Third cycle** | **Fourth cycle** | **Fifth cycle** | **Sixth cycle** | **Duration of therapy**  **(short-term vs. prolonged)** | **Outcome** |
| --- | --- | --- | --- | --- | --- | --- | --- | --- |
| **UAE 12** | Terbinafine, 250 mg, PO, daily, 42 days + ketoconazole topical 2% cream, BID, 28 days + ketoconazole topical 2% shampoo, daily 30 days | — | — | — | — | — | prolonged-6 weeks | **No follow up** |
| **UAE 21** | terbinafine oral, 125 mg, PO, Daily, 6 weeks | terbinafine oral; 250 mg, PO, Daily, 6 weeks+terbinafine topical 1% spray; 1 spray, BID, 60 days Nizoral topical shampoo; 1 app, Shampoo, TOP, 2x/wk, 60 days | — | — | — | — | prolonged-15 weeks | **Recurrence** |
| **UAE 50** | ketoconazole topical cream, BID, 28 days | — | — | — | — | — | short - 4 weeks | **Improved** |
| **UAE 57** | ketoconazole topical 2% shampoo, daily, 3 weeks. | Terbinafine, 62.5 mg, PO, daily, 30 days + ketoconazole topical 2% shampoo, daily, 1 month | — | — | — | — | prolonged 7 weeks | **Recurrence** |
| **UAE 58** | Terbinafine topical 1% cream,1 app, BID, 21 days | — | — | — | — | — | short - 3 weeks | **No follow up** |
| **UAE 65** | Terbinafine, 62.5 mg, PO, daily, 8 weeks + ketoconazole topical 2% shampoo, 1 app, Shampoo, TOP, 3x/week, 8 weeks | — | — | — | — | — | prolonged - 8 weeks | **No follow up** |
| **UAE 66** | Terbinafine, 62.5 mg, PO, daily, 8 weeks + ketoconazole topical 2% shampoo, 1 app, Shampoo, TOP, 3x/week, 8 weeks | — | — | — | — | — | prolonged - 8 weeks | **Recurrence** |
| **UAE 80** | Clotrimazole topical 1% cream, 1 app, BID, 7 days | — | — | — | — | — | Short - 1 week | **No follow up** |
| **UAE 103** | on fluconazole as antifungal prophylaxis. clotrimazole topical 1% cream; 1 app, Cream, TOP, BID + ketoconazole topical 2% shampoo; 1 app, Shampoo, TOP, MTh,J14 + terbinafine oral; 250 mg, PO, Daily, 14 day(s) | — | — | — | — | — | short - 2 weeks | **No follow up** |
| **UAE 105** | Terbinafine topical 1% cream,1 app, BID, 30 days | — | — | — | — | — | Prolonged - 5 weeks | **No follow up** |
| **UAE 106** | Fluconazole, 50 mg, 7days, soft stop + terbinafine topical 1% cream: 1 app, BID, 30 days | Terbinafine oral, 125 mg, PO, Daily + ketoconazole topical, 1 app, Shampoo, TOP, 2x/wk, + terbinafine topical, 1 app, Cream, BID( 30 days) | — | — | — | — | Prolonged - 9 weeks | **Recurrence** |
| **UAE 116** | Clotrimazole cream + ketoconazole shampoo. | Itraconazole, 100 mg, PO, BID, 14 days (this dose actually taken onyl for 1 week before dose change) + ketoconazole topical, 1 app, Shampoo, TOP, 2x/wk, 30 days + terbinafine topical, 1 spray, Spray, TOP, BID, 14 days | itraconazole, 200 mg, PO, BID | itraconazole, 200 mg, PO, BID, 30 days + ketoconazole topical, 1 app, cream, TOP, BID, 14 days + terbinafine topical, 1 spray, Spray, TOP, BID, 14 days. | itraconazole, 100 mg, PO, BID, 14 days + ketoconazole topical, 1 app, Shampoo, TOP, 2x/wk, 30 days + terbinafine topical, 1 spray, Spray, TOP, BID, 14 days + ketoconazole topical, 1 app, cream, TOP, BID, 14 days | itraconazole, 100 mg, PO, BID, 14 days + topicals | Prolonged - 7 weeks | **Recurrence** |
| **UAE 142** | Ketoconazole topical 2% shampoo, 1 app, daily for 1 week then 2x/week, 6 weeks + clotrimazole topical 1% cream, 1 app, BID, for 30 days | Ketoconazole topical 2% shampoo, 1 app, daily for 1 week then 2x/week, 6 weeks + clotrimazole topical 1% cream, 1 app, BID, for 30 days | — | — | — | — | Prolonged – 9 weeks | **Improved** |
| **UAE 183** | Oral Terbinafine two full courses., Fluconazole 70 mg daily. , Topical Terbinafine spray, Luliconazole and clotrimazole creams, Ketoconazole shampoo. | — | — | — | — | — | prolonged-21 weeks | **Improved** |
| **UAE 189** | Clotrimazole topical 1% cream, 1 application, BID, 3 weeks | Itraconazole oral, 100 mg, BID, 14 days + terbinafine topical 1% cream, 1 application, BID, 21 days + clotrimazole topical 1% cream, 1 application, BID, 3 weeks | — | — | — | — | prolonged - 6 weeks | **Recurrence** |
| **UAE 190** | Fluconazole, 75 mg, PO, daily, 21 days + ketoconazole topical 2% shampoo, 1 app, 2x/week, 60 days | Fluconazole, 75 mg, PO, daily, 21 days + ketoconazole topical 2% shampoo, 1 app, 2x/week, 60 days. | Fluconazole, 75 mg, PO, daily, 21 days + ketoconazole topical 2% shampoo, 1 app, 2x/week, 30 days | ketoconazole topical 2% shampoo, 1 app, 2x/week, 90 days. | — | — | prolonged - 30 weeks | **Recurrence** |
| **UAE 228** | Ketoconazole topical 2% shampoo, 1 app, q7day, 14 days | Terbinafine oral, 62.5 mg, PO, Daily, 30 days + terbinafine topical, 1 spray, BID, 30 days + ketoconazole topical 2% shampoo, 1 app, 2x/wk, 30 days. | Terbinafine oral, 62.5 mg, PO, Daily, 30 days + terbinafine topical, 1 spray, BID, 30 days + ketoconazole topical 2% shampoo, 1 app, 2x/wk, 30 days | Terbinafine oral, 62.5 mg, PO, Daily, 30 days + terbinafine topical, cream, daily, 30 days + ketoconazole topical 2% shampoo, 1 app, 2x/wk, 30 days + clotrimazole topical, 1 app, Daily, for 30 days | ketoconazole topical 2% shampoo, 1 app, 2x/wk, 21 day | — | Prolonged - 17 weeks | **Recurrence** |
| **UAE 239** | mometasone 0.1% cream - ketaconazole 2% cream, used for 10 days or until infection resolves | ketoconzaole topical 1% cream, BID, 30 days | Itraconzaole, 50 mg, PO, daily, 30 day (NOT TAKEN DUE TO BAD TASTE) + ketoconzaole topical 1% cream, BID, 30 days | terbinfaine, 62.5 mg, PO, daily, 30 days | — | — | Prolonged - 14 weeks | **Recurrence** |
| **UAE 243** | clotrimazole topical 1% cream, topical, BID, 3 weeks + ketoconazole topical 2% shampoo, 2x / week, 30 days | — | — | — | — | — | Prolonged - 5 weeks | **Improved** |
| **UAE 263** | Floconazole, 50 mg, PO, daily, 21 days + terbiafine topical, BID, 30 days + ketoconazole topical, 3x / week, 30 days | Terbinafine topical 1%, daily, 30 days + ketoconazole topical 2% shampoo, daily, soft stop + clotrimazole topical 1% cream, BID, 7 days + LamISIL (terbinfaine) 1% topical spray, 1 spray, BID, 7 days | — | — | — | — | Prolonged - 9 weeks | **Recurrence** |
| **UAE 266** | Terbinafine oral, 125 mg, PO, daily, 30 days + ketoconazole topical, 1 application, daily, 30 day | Terbinafine oral, 125 mg, PO, daily, 30 days + ketoconazole topical, 1 application, daily, 30 days | Terbinfaine oral, 125 mg, PO, daily 14 days + ketoconazole topical, 1 application, daily, 30 days. | — | — | — | Prolonged – 13 weeks | **Recurrence** |
| **UAE 291** | Terbinafine oral, 62.5 mg, PO, daily, 42 days + ketoconazole topical shampoo, q72 hours, 1 month | Terbinafine oral, 62.5 mg, PO, daily, 30 days + ketoconazole topical cream, 1 application, daily, 30 days | — | — | — | — | Prolonged - 10 weeks | **Recurrence** |
| **UAE 315** | Clotrimazole topical, 1 app, TID, 21 days | — | — | — | — | — | short - 3 weeks | **No follow up** |
| **UAE 320** | betamethasone-fusidic acid cream | — | — | — | — | — | — | **No follow up** |
| **UAE 321** | clotrimazole topical, 1 app, BID, 14 days | — | — | — | — | — | short - 2 weeks | **No follow up** |
| **UAE 322** | Terbinafine 1% cream, 1 app, BID, 2 weeks | — | — | — | — | — | short - 2 weeks | **Improved** |
| **UAE 333** | Clotrimazole topical 1% cream, BID, 7 days + ketoconazole topical 2% shampoo, 2x/week, 14 days | Terbinafine, 125 mg, PO, daily, 30 days | Terbinafine, 62.5 mg, PO, daily 30 days + ketoconazole topical cream, daily, 40 days. | — | — | — | Prolonged - 12 weeks | **Recurrence** |
| **UAE 334** | Terbinafine, 62.5 mg, PO, 30 days + ketoconazole topical 2% cream, daily, 30 days | — | — | — | — | — | Prolonged - 5 weeks | **No follow up** |
| **UAE 340** | Fluconazole, 75 mg, PO, q7day, for 28 days + ketoconazole topical, 1 app, TOP, 2x/week, 30 days + terbinafine topical, 1 spray, BID, 30 days. | Terbinafine, 62.5 mg, PO, Daily, 6 weeks | — | — | — | — | Prolonged - 11 weeks | **Recurrence** |
| **UAE 341** | Terbinafine, 125 mg, PO, Daily, 6 weeks + amoxicillin-clavulanate, 790 mg, PO, q12hr, 7 days + clindamycin, 300 mg, PO, TID, 7 days | — | — | — | — | — | Prolonged - 6 weeks | **No follow up** |
| **UAE 342** | Itraconazole, 50 mg, PO, daily, 4 weeks + ketoconazole 2% shampoo, 2x/week, 30 days + terbinafine hydrochloride 1% spray, 5 puffs, BID, 4 weeks + luliconazole 1% cream, 1 app, daily, 4 weeks | ketoconazole topical 2% shampoo, alternate day, 14 days | Terbinafine, 62.5 mg, PO, Daily, 6 weeks | — | — | — | Prolonged - 12 weeks | **Recurrence** |
| **UAE 355** | None | — | — | — | — | — | — | **Unknown** |
| **UAE 356** | Terbinafine, 62.5 mg, PO, Daily, 30 days + ketoconazole topical 2% cream, daily, 30 days | Terbinafine, 62.5 mg, PO, Daily, 30 days + ketoconazole topical 2% cream, daily, 30 days | — | — | — | — | Prolonged – 9 weeks | **Recurrence** |
| **UAE 357** | Clotrimazole topical, 1 app, TOP, BID, 30 days + ketoconazole topical, 1 app, TOP, daily for 1 week then 2x/week, 6 weeks | — | — | — | — | — | Prolonged - 6 weeks | **No follow up** |
| **UAE 368** | Ketoconazole topical, 1 app, TOP, 3x/week, 1 month | — | — | — | — | — | Prolonged - 5 weeks | **No follow up** |
| **UAE 369** | Clotrimazole topical: 1 app, TOP, BID, 30 days + ketoconazole topical, 1 app, TOP, 2x/week, 30 days + terbinafine oral: 125 mg, PO, Daily, 30 days (only 14 days dispensed) | Terbinafine oral 62.5 mg, PO, Daily, 8 weeks (not dispensed) + continue previous medications | Clotrimazole topical: 1 app, TOP, BID, 30 days + ketoconazole topical, 1 app, TOP, 2x/week, 30 days + terbinafine oral: 26.5 mg, PO, Daily, 30 days (not dispensed) | Clotrimazole topical: 1 app, TOP, BID, 6 weeks + ketoconazole topical, 1 app, TOP, 2x/week, 6 weeks + fluconazole, 150 mG, oral, q7days, 6 weeks (no medications dispensed) | — | — | Prolonged - 24 weeks | **Recurrence** |
| **UAE 390** | Ketoconazole topical, 1 app, TOP, daily for 2 weeks then alternate days for 2 weeks then 2x/week as maintinance + terbinafine oral, 187.5 mg, PO, Daily, 6 weeks | — | — | — | — | — | prolonged - 6 weeks | **No follow up** |
| **UAE 395** | fluconazole: 50 mg, PO, q7day, 60 days + ketoconazole topical: 1 app, TOP, 2x/week, 1 month + terbinafine topical, 1 app, TOP, BID, 14 days | nystatin topical, 1 app, TOP, BID, 14 days + continuation of previous medications | — | — | — | — | Prolonged - 11 weeks | **Recurrence** |
| **UAE 396** | Terbinafine topical: 1 app, TOP, BID, for 14 day(s) | Terbinafine oral: 62.5 mg, PO, Daily, for 45 day(s) + continue terbinafine cream | Clotrimazole topical: 1 app, TOP, BID, for 14 day(s) + itraconazole: 75 mg, PO, Daily, for 4 week(s) + ketoconazole topical: 1 app, TOP, 2x/week, for 30 day( | — | — | — | prolonged - 13 weeks | **Recurrence** |
| **UAE 397** | Ketoconazole topical: 1 app, TOP, daily for 1 week then twice per week, 6 weeks + terbinafine oral: 62.5, PO, Daily, for 30 day | — | — | — | — | — | Prolonged - 6 weeks | **No follow up** |
| **UAE 268** | Ketoconazole topical 2% cream, 1 app, Topical, Daily, 30 days + terbinafine oral, 62.5 mg, PO, Daily, 30 days | Ketoconazole topical, 1 app, Shampoo, TOP, 2x/week, 30 day | Ketoconazole topical 2% cream, 1 app, Topical, Daily, 21 days | — | — | — | Prolonged – 12 weeks | **Recurrence** |
| **UAE 423** | Terbinafine oral: 125 mg, PO, Daily, for 6 weeks. | Clotrimazole topical: 1 app, TOP, BID, for 30 day(s).ketoconazole topical: 1 app, TOP, MTh, for 30 day(s). | Fluconazole: 150 mg, PO, q7day, for 6 week(s),ketoconazole topical: 1 app, TOP, SuTu, for 45 day(s),ketoconazole topical: 1 app, TOP, MTh, for 30 day(s),luliconazole topical: 1 app, Topical, Daily, for 45 day(s). | Ketoconazole topical 2% cream, 1 app, Topical, Daily, 21 days + Ketoconazole topical 2% shampoo, 1 app, Topical, q7day, 90 days" | — | — | Prolonged - 29 weeks | **Recurrence** |
| **UAE 424** | Ketoconazole topical: 1 app, TOP, SuW, for 60 day(s) 2 bottle, 1 Refill(s).terbinafine topical: 1 app, TOP, BID, for 30 day(s). | :Fluconazole: 50 mg, PO, Daily, for 30 day(s). topical: 1 app, TOP, SuW, for 60 day(s), 2 bottle, 1 Refill(s).terbinafine topical: 1 app, TOP, BID, for 30 day(s). | — | — | — | — | Prolonged - 10 weeks | **Recurrence** |
| **UAE 436** | Clotrimazole topical: 1 app, TOP, BID, for 30 day(s).fluconazole: 150 mg, PO, Daily, for 28 day(s). | Itraconazole oral liquid, 100 mg, PO, Daily ketoconazole topical 2% cream, 1 app, Topical, BID ketoconazole topical 2% shampoo, 1 app, Topical | — | — | — | — | Prolonged - 4 weeks + | **Recurrence** |
| **UAE 443** | Fluconazole: 50 mg, PO, q7day, for 60 day(s), 8 cap. hydrogen peroxide: 1 mL, Topical, Daily, for 5 day(s), 5 mL. ketoconazole topical: 1 app, TOP, SuTh, for 1 month(s). nystatin topical: 1 app, TOP, TID, for 7 day(s), 1 tube. terbinafine topical: 1 app, TOP, BID, for 7 day(s), 1 tube. | — | — | — | — | — | prolonged - 9 weeks | **Improved** |
| **UAE 452** | Ketoconazole topical: 1 app, TOP, daily, for 3 weeks. terbinafine topical: 1 app, TOP, BID, for 21 days | Clotrimazole topical: 1 app, TOP, daily, for 4 week(s). fluconazole: 200 mg, PO, q7day, for 4 week(s). ketoconazole topical: 1 app, TOP, daily, for 3 weeks. ketoconazole topical: 1 , TOP, 3x/week, for 4 weeks. terbinafine topical: 1 app, TOP, BID, for 4 weeks(s)." | — | — | — | — | Prolonged - 7 weeks | **Recurrence** |
| **UAE 458** | Fluconazole 6 mg/kg, PO, daily, 14 days | Terbinafine, 125 mg, PO, daily, 30 days ketoconazole topical: 1 app, TOP, BID, for 28 days. ketoconazole topical: 1 app, TOP, 2x/week, for 30 days | — | — | — | — | Prolonged - 7 weeks | **Recurrence** |
| **UAE 469** | Clotrimazole topical: 1 app, TOP, BID, for 7 days. | Terbinafine, 125 mg, PO, daily, 7 days. clotrimazole topical: 1 app, TOP, BID, for 7 days. ketoconazole topical: 1 app, TOP, 2x/week, for 14 days. | — | — | — | — | short-3 weeks | **Recurrence** |

**Outcome key:** ■ **Improved**	■ **Recurrence**	■ **No follow up / Unknown**

*Abbreviations: PO, per os (oral); TOP, topical; BID, twice daily; TID, three times daily; q7day, once weekly; q12hr, every 12 hours; app, application; 2x/wk, twice weekly.*
