# Supplementary material for: Microsporum canis complex infections in the United Arab Emirates: Clinical, molecular, and antifungal susceptibility profiles
Source: Med Mycol. 2026 Aug 29;64(9):myag091. doi: 10.1093/mmy/myag091 (PMC13570593; doi:10.1093/mmy/myag091)
Supplement: Supplementary file 1 [file myag091_supplemental_files.zip › mm-2026-0224-File009.docx]

**Supplementary Table S2.** Strain-wise macroscopic and microscopic characteristics of *M. canis* complex (*n*=52) isolates, with representative images and adjunct test findings.

| **Isolate ID** | **Species / Molecular ID** | **Macromorphology (PDA)** | | **Micromorphology (LPCB* / 10% LA* mounts)** | **Adjunct Physiological tests** | **Unique features** |
| --- | --- | --- | --- | --- | --- | --- |
| **UAE 12** | ***M. audouinii*** |  | |  |  | Macroconidia abundant, mostly spindle-shaped with some bizarre forms; thick, rough, multi-septate. Microconidia sparse, small, ovoid–clavate; occasional chlamydoconidia present. |
|  |  |  |  |  |  |  |
|  |  |  | |  |  |  |
| **UAE 333** | ***M. audouinii*** |  | |  |  | Macroconidia abundant, indistinguishable from  *M. canis*, spindle-shaped, thick-walled, and multiseptate, with occasional distorted forms; pectinate hyphae evident. Microconidia club- to clavate-shaped; chlamydoconidia not observed. |

| **UAE 403** | ***M. audouinii***  |  | | | |  | |  | Pectinate hyphae and chlmydoconidia prominent; macroconidia sparse and often bizarre or distorted rather than classical, with irregular, elongated forms.  Microconidia present as small lateral elements; no well-formed typical  *M. canis*-like macroconidia seen. |
| --- | --- | --- | --- | --- | --- | --- | --- | --- | --- |
|  |  |  |  |  |  |  |  |  |  |
|  |  |  |  |  |  |  |  |  |  |
|  |  |  |  |  |  |  |  |  |  |
|  |  |  | | | |  | |  |  |

| **UAE 440** | ***M. audouinii***  |  |  |  | Pectinate hyphae prominent with abundant chlamydoconidia in chains, intercalary and terminal; macroconidia very sparse, smooth-walled, and atypical.  Overall features consistent with classical  *M. audouinii*. |
| --- | --- | --- | --- | --- | --- |
| **UE 452** | ***M. audouinii*** |  |  |  | Pectinate hyphae present with fewer chlamydoconidia; macroconidia very sparse and predominantly bizarre (elongated forms), distorted rather than classical, with occasional smooth-walled forms.  Disintegrated hair on HPT. |

| **UAE 355** | ***M. canis (distortum)*** |  |  | |  | Abundant, thick-walled, rough, spindle-shaped macroconidia typical of  *M. canis*, with numerous contorted/distorted (“distortum”) forms; microconidia present along hyphae. Morphology consistent with *M. canis* variant. |
| --- | --- | --- | --- | --- | --- | --- |
|  |  |  |  |  |  | Extensive perforations and breaking of hair in HPT. |
| **UAE 21** | ***M. canis*** |  |  | | Not performed | Thick-walled, rough, spindle-shaped macroconidia typical of  *M. canis* seen. Failed to revive from -80^o^C |
|  |  |  |  |  |  |  |
|  |  |  |  | |  |  |

| **UAE 50** | ***M. canis*** |  |  |  | Moderate Thick-walled, rough, tapering to spindle-shaped macroconidia observed. |
| --- | --- | --- | --- | --- | --- |
|  |  |  |  |  |  |
|  |  |  |  |  |  |

| **UAE 183** | ***M. canis*** |  |  |  | Distortum-like features: Macroconidia moderately present, showing intermediate features with a mix of spindle-shaped and distorted forms; walls variably thick and occasionally roughened, bridging *M. canis*.  Distortum, and *M. audouinii*-like morphologies. Pectinate hyphae seen. |
| --- | --- | --- | --- | --- | --- |
|  |  |  |  |  |  |
|  |  |  |  |  |  |
| **UAE 57** | ***M. canis*** |  |  |  | Macroconidia present, showing transition from typical spindle-shaped forms to contorted/distorted *M. canis*-like morphology, with thickened walls and developing irregular outlines. |
|  |  |  |  |  |  |
|  |  |  |  |  |  |

| **UAE 65** | ***M. canis*** |  |  |  | Few chlamydoconidia, pectinate and arthroconidial hyphae seen ; moderate macroconidia with a few bizarre, distorted forms and few smooth forms and others were consistent with *M. canis* morphology observed. Microconidia pyriform and borne laterally along hyphae. Urease activity was weak. |
| --- | --- | --- | --- | --- | --- |
| **UAE 58** | ***M. canis*** |  |  |  | Numerous chlamydoconidia, often in chains; moderate macroconidia present with bizarre, distorted forms and few were consistent with *M. canis* morphology.  Microconidia pyriform and borne laterally along hyphae. |
|  |  |  |  |  |  |
|  |  |  |  |  |  |

| **UAE 66** | ***M. canis*** |  |  |  | Prominent pectinate hyphae seen; moderate macroconidia with a few distorted forms and others consistent with *M. canis* morphology observed. |
| --- | --- | --- | --- | --- | --- |
| **UAE 80** | ***M. canis*** |  |  |  | Abundant macroconidia, thick-walled, coarsely roughened, spindle- to canoe-shaped with multiple septa and characteristic rostrate/bent apices with basal frill. Microconidia sparse, lateral, unicellular, smooth-walled, ovoid to clavate and nonspecific. |
|  |  |  |  |  |  |
|  |  |  |  |  |  |

| **UAE 103** | ***M. canis*** |  |  |  | No pigmentation on PDA, sparse conidiation. Weak HPT results.  Poorly conidiating strain with predominantly sterile, septate, hyaline hyphae; macroconidia rare and confined to occasional sectors. |
| --- | --- | --- | --- | --- | --- |
| **UAE 105** | ***M. canis*** |  |  |  | Distortum-like features seen. Chlamydoconidia present along hyphae as thick-walled rounded cells; macroconidia spindle-shaped, multiseptate with some irregular forms, and microconidia sparse and lateral. |

| **UAE 106** | ***M. canis*** |  |  |  | Abundant macroconidia, spindle-shaped, thick-walled, and multiseptate with roughened surfaces and pointed/rostrate apices; typical *M. canis* morphology.  Microconidia sparse and inconspicuous. |
| --- | --- | --- | --- | --- | --- |
|  |  |  |  |  |  |
|  |  |  |  |  |  |
| **UAE 116** | ***M. canis*** |  |  |  | Numerous arthroconidial hyphae evident; macroconidia present with numerous bizarre, distorted shapes rather than classical forms. |
|  |  |  |  |  |  |
|  |  |  |  |  |  |

| **UAE 142** | ***M. canis*** |  |  |  | Rare features include intercalary chlamydoconidia, pectinate hyphae, and spherical microconidia; macroconidia are few. No pigmentation observed on rice grain or PDA. |
| --- | --- | --- | --- | --- | --- |
|  |  |  |  |  |  |
|  |  |  |  |  |  |
| **UAE 189** | ***M. canis*** |  |  |  | Typical *M. canis* features with thick-walled, rough, spindle-shaped multiseptate macroconidia and sparse lateral microconidia.  This strain shows long, tapering macroconidia (often >12 septa) with occasional distorted forms; cultures initially sterile with conidiation limited to few sectors. |

| **UAE 190** | ***M. canis*** |  |  |  | Negative HPT results. Abundant, classical *M. canis* macroconidia, echinulate, spindle-shaped and multiseptate with a characteristic lateral beak; occasional macroconidia show distorted lateral tips.  Microconidia sparse. |
| --- | --- | --- | --- | --- | --- |
|  |  |  |  |  |  |
|  |  |  |  |  |  |
| **UAE 228** | ***M. canis*** |  |  |  | Abundant, classical *M. canis* macroconidia, spindle-shaped, echinulate, multiseptate with pointed apices and characteristic tapering ends. Microconidia sparse. |
|  |  |  |  |  |  |
|  |  |  |  |  |  |

| **UAE 239** | ***M. canis*** |  | |  |  | Urease activity variable to weak. Rare features include presence of chlamydoconidia, along with pectinate and arthroconidial hyphae; macroconidia are few. No pigmentation observed on rice grain or PDA. |
| --- | --- | --- | --- | --- | --- | --- |
|  |  |  | |  |  |  |
|  |  |  | |  |  |  |
| **UAE 243** | ***M. canis*** |  | |  |  | No pigment on PDA. Abundant, classical *M. canis* macroconidia, spindle-shaped, thick-walled, coarsely roughened, and multiseptate with pointed/tapering apices; well-formed typical morphology, seen mostly on dense cottony sectors of the colony.  Microconidia sparse and lateral. |
|  |  |  |  |  |  |  |
|  |  |  | |  |  |  |

| **UAE 263** | ***M. canis*** |  | |  |  | Abundant macroconidia, spindle-shaped, thick-walled, and multiseptate with prominent tapering ends; morphology consistent with a tapering *M. canis* strain. Microconidia sparse. |
| --- | --- | --- | --- | --- | --- | --- |
|  |  |  |  |  |  |  |
|  |  |  | |  |  |  |
| **UAE 266** | ***M. canis*** |  | |  |  | Typical *M. canis* features with spindle-shaped, thick-walled, multiseptate macroconidia; additionally, **rare 2-celled macroconidia observed.**  Arthroconidial hyphae and chlamydoconidia also present. |
|  |  |  | |  |  |  |
|  |  |  | |  |  |  |

| **UAE 268** | ***M. canis*** *(received from referral laboratory as*  *T. rubrum)* |  |  |  | Initially submitted by the referral laboratory as *Trichophyton rubrum*.  Predominantly sterile, poorly conidiating isolate; repeated slide cultures showed mainly septate hyaline hyphae with chlamydoconidia and pectinate hyphae, whereas tape mounts from rare conidiating sectors on sterile rice grain revealed characteristic *M. canis* macroconidia and sparse microconidia. HPT was variable, urease negative, and no pigment was produced on rice grain or PDA; only a faint  orange-cinnamon reverse developed on SDA-C after prolonged incubation. |
| --- | --- | --- | --- | --- | --- |

| **UAE 291** | ***M. canis*** |  |  |  | Abundant, classical *M. canis* macroconidia, spindle-shaped, thick-walled, coarsely roughened and multiseptate with pointed/tapering apices; well-formed typical morphology.  Microconidia sparse and lateral. No pigmentation on rice grain and HPT was variable. |
| --- | --- | --- | --- | --- | --- |
| **UAE 314** | ***M. canis*** |  |  |  | Classical *M. canis* morphology with spindle-shaped, thick-walled, coarsely roughened, multiseptate macroconidia with tapering ends.  Microconidia numerous, smooth-walled, ovoid to club shaped, borne laterally along hyphae. |

| **UAE 315** | ***M. canis*** |  |  |  | Abundant, classical *M. canis* macroconidia, spindle-shaped, thick-walled, coarsely roughened and multiseptate with tapering apices; few bizarre/distorted forms also present.  Microconidia sparse. |
| --- | --- | --- | --- | --- | --- |
| **UAE 320** | ***M. canis*** |  |  |  | Poorly conidiating strain with prominent arthroconidial hyphae and numerous club-shaped microconidia; macroconidia scarce and infrequently observed. No pigmentation on rice grain was observed. |
|  |  |  |  |  |  |
|  |  |  |  |  |  |

| **UAE 321** | ***M. canis*** |  | |  |  | Classical *M. canis* morphology with spindle-shaped, thick-walled, coarsely roughened, multiseptate macroconidia with tapering apices; well-formed typical features. Microconidia sparse and lateral. Disintegration of hair on HPT. |
| --- | --- | --- | --- | --- | --- | --- |
|  |  |  |  |  |  |  |
|  |  |  | |  |  |  |
| **UAE 322** | ***M. canis*** |  | |  |  | Poorly conidiating strain with sparse macroconidia restricted to some sectors; arthroconidial forms predominate.  Microconidia numerous, club-shaped and borne along hyphae. |

| **UAE 340** | ***M. canis*** |  |  |  | Classical *M. canis* morphology with abundant spindle-shaped, thick-walled, multiseptate macroconidia; notably long, tapering forms (>13 cells) present.  Arthroconidial forms and racquet hyphae seen, with sessile microconidia borne on undifferentiated hyphae. |
| --- | --- | --- | --- | --- | --- |
| **UAE 341** | ***M. canis*** |  |  |  | Classical *M. canis* macroconidia in clusters, spindle-shaped, thick-walled and multiseptate; many show bizarre shapes with clumped material on their surface. Numerous racquet hyphae present. |

| **UAE 334** | ***M. canis*** |  |  |  | Sparse conidiation with few macroconidia, which are spindle-shaped, multiseptate, and lightly roughened; microconidia inconspicuous. No pigmentation observed on rice grain. |
| --- | --- | --- | --- | --- | --- |
|  |  |  |  |  |  |
|  |  |  |  |  |  |
| **UAE 342** | ***M. canis*** |  |  |  | Sparse conidiation with few macroconidia, which are spindle-shaped, multiseptate, and lightly roughened; microconidia club shaped. |
|  |  |  |  |  |  |
|  |  |  |  |  |  |

# UAE 356

## M. canis

Classical *M. canis* morphology with abundant spindle-shaped, thick-walled, multiseptate macroconidia observed.

# UAE 357

## M. canis

Classical *M. canis* morphology with abundant spindle-shaped, thick-walled, multiseptate macroconidia observed.

| **UAE 358** | ***M. canis*** |  |  |  | Classical *M. canis* morphology with abundant spindle-shaped, thick-walled, multiseptate macroconidia with prominent terminal beak observed. |
| --- | --- | --- | --- | --- | --- |
|  |  |  |  |  |  |
|  |  |  |  |  |  |

| **UAE 368** | ***M. canis*** |  |  |  | Abundant macroconidia with prominent lateral beak, (parrot-like) spindle-shaped and multiseptate, showing distinctly echinulate (rough, spiny) surface projections; morphology typical of *M. canis*. |
| --- | --- | --- | --- | --- | --- |
|  |  |  |  |  |  |
|  |  |  |  |  |  |

***M. canis***

Classical *M. canis* morphology with moderate spindle-shaped, thick-walled, multiseptate macroconidia were observed.

**UAE 369**

| **UAE 390** | ***M. canis*** |  |  |  | Abundant, classical *M. canis* macroconidia, spindle-shaped, thick-walled, coarsely roughened and multiseptate with tapering/pointed apices; well-formed typical morphology.  Microconidia sparse and lateral. |
| --- | --- | --- | --- | --- | --- |
|  |  |  |  |  |  |
|  |  |  |  |  |  |
| **UAE 395** | ***M. canis*** |  |  |  | Sparse conidiation with few macroconidia; tapering macroconidia present.  Chlamydoconidia and pectinate hyphae also observed. |
|  |  |  |  |  |  |
|  |  |  |  |  |  |

| **UAE 396** | ***M. canis*** |  |  |  | Macroconidia predominantly tapering, spindle-shaped and multiseptate, with a few showing distortum-like curved/irregular forms; overall morphology consistent with *M. canis*. Microconidia sparse. |
| --- | --- | --- | --- | --- | --- |
|  |  |  |  |  |  |
|  |  |  |  |  |  |
| **UAE 397** | ***M. canis*** |  |  |  | Macroconidia predominantly tapering, spindle-shaped, thick-walled and multiseptate, with occasional distorted/irregular forms; consistent with *M. canis*. Microconidia sparse. |

***M. canis***

Classical *M. canis* morphology with abundant spindle-shaped, thick-walled, multiseptate macroconidia observed.

**UAE 423**

# UAE 424

## M. canis

Macroconidia showing transition from typical spindle-shaped forms to distorted *M. canis*-distortum like morphology. Raquet and thick hyphae are prominent in this strain.

# UAE 436

## M. canis

Classical *M. canis* morphology with abundant spindle-shaped, thick-walled, multiseptate macroconidia observed. Clavate to pyriform microconidia seen.

| **UAE 443** | ***M. canis*** |  | |  |  | Classical *M. canis* morphology with abundant spindle-shaped, thick-walled, coarsely roughened, multiseptate macroconidia with tapering/pointed apices; well-developed typical forms. Microconidia numerous, pyriform to club shaped, borne laterally along hyphae. |
| --- | --- | --- | --- | --- | --- | --- |
| **UAE 458** | ***M. canis*** |  | |  |  | Classical *M. canis* morphology with spindle-shaped, thick-walled, coarsely roughened, multiseptate macroconidia showing tapering apices; well-formed typical structures. Microconidia numerous, small, club shaped, and borne laterally along hyphae. |
|  |  |  |  |  |  |  |
|  |  |  | |  |  |  |

| **UAE 469** | ***M. canis*** |  |  |  | Sparse conidiation with few macroconidia, some showing distorted/irregular forms; Morphology consistent with *M. canis*.  Microconidia inconspicuous. |
| --- | --- | --- | --- | --- | --- |

Footnotes: PDA = Potato Dextrose Agar; LPCB = Lactophenol Cotton Blue; LA = Lactic acid.

**Overall description of *M canis* strains morphology**: Microconidia, when present, arose laterally from vegetative hyphae by holoblastic conidiogenesis and were produced singly and asynchronously. They were unicellular, aseptate, hyaline, smooth-walled, and ovoid to clavate, borne sessile or on short lateral projections, and were morphologically nonspecific. Macroconidia constituted the principal diagnostic feature and were abundantly produced in most isolates. They arose from fertile hyphae via holothallic, holoblastic conidiogenesis, occurring singly or in small groups at acute angles, with schizolytic dehiscence. Macroconidia were hyaline, thick-walled, coarsely roughened, spindle- to canoe-shaped, measuring ~35–110 × 12–25 µm with 5–15 cells (commonly 6–12). The apical cell was often rostrate or bent, frequently with a verrucose knob, and a basal frill was usually present. For poorly sporulating isolates (e.g., UAE268), representative images from repeated slide cultures and tape mounts of localized conidiating sectors are shown to illustrate the development of diagnostic morphological structures.
